# Mechanically Robust and Flame-Retardant Polylactide Composites Based on In Situ Formation of Crosslinked Network Structure by DCP and TAIC

**DOI:** 10.3390/polym14020308

**Published:** 2022-01-13

**Authors:** Yajun Chen, Xingde Wu, Mengqi Li, Lijun Qian, Hongfu Zhou

**Affiliations:** 1 School of Chemical and Materials Engineering, Beijing Technology and Business University, Beijing 100048, China; w13014634514@163.com (X.W.); limengqi202009@163.com (M.L.); zhouhongfu@th.btbu.edu.cn (H.Z.); 2 China Light Industry Advanced Flame Retardant Engineering Technology Research Center, Beijing 100048, China; 3 Petroleum and Chemical Industry Engineering Laboratory of Non-Halogen Flame Retardants for Polymers, Beijing 100048, China

**Keywords:** poly(lactic acid), crosslinked structures, mechanical property, flame retardant property, crystallization behavior

## Abstract

The addition of intumescent flame retardant to PLA can greatly improve the flame retardancy of the material and inhibit the dripping, but the major drawback is the adverse impact of the mechanical properties of the material. In this study, we found that the flame retardant and mechanical properties of the materials can be improved simultaneously by constructing a cross-linked structure. Firstly, a cross-linking flame-retardant PLA structure was designed by adding 0.9 wt% DCP and 0.3 wt% TAIC. After that, different characterization methods including torque, melt flow rate, molecular weight and gel content were used to clarify the formation of crosslinking structures. Results showed that the torque of 0.9DCP/0.3TAIC/FRPLA increased by 307% and the melt flow rate decreased by 77.8%. The gel content of 0.9DCP/0.3TAIC/FRPLA was 30.8%, indicating the formation of cross-linked structures. Then, the mechanical properties and flame retardant performance were studied. Results showed that, compared with FRPLA, the tensile strength, elongation at break and impact strength of 0.9DCP/0.3TAIC/FRPLA increased by 34.8%, 82.6% and 42.9%, respectively. The flame retardancy test results showed that 0.9DCP/0.3TAIC/FRPLA had a very high LOI (the limiting oxygen index) value of 39.2% and passed the UL94 V-0 level without dripping. Finally, the crosslinking reaction mechanism, flame retardant mechanism and the reasons for the improvement of mechanical properties were studied and described.

## 1. Introduction

With the growing awareness of sustainability in public perception, and more regulations restricting the materials being used in various of applications, biodegradable materials have become the focus in many fields [1,2,3,4]. PLA has attracted much attention due to its biodegradability, excellent mechanical performance, good processing capability and acceptable cost. However, flammability and heavy dropping behavior during combustion greatly limit the application of PLA in electronic and electrical appliances, rail transit and other fields [5,6,7]. Therefore, researchers have conducted enormous in-depth studies on the flame-retardant PLA. At present, the flame retardant system can be generally categorized into phosphorus containing flame retardants [8,9,10], nano-flame retardants [11,12,13], polymer molecules (hyperbranched polymer, etc.) [14,15] and metal hydroxide flame retardants [16]. The introduction of the flame retardant system can effectively inhibit the flammability of PLA. However, as a filler in PLA composite system, the flame retardant system greatly reducing the mechanical properties of the composites. Therefore, enhancing the flame retardant properties with maintained or improved mechanical properties of PLA has become the focus of current research.

In recent years, some researchers improved the comprehensive properties of flame-retardant PLA with the addition of a toughening agent [17]. Such as the thermoplastic polyurethane elastomer (TPU) [18,19], crosslinked polyurethane (CPU) [20], biobased unsaturated polyester (BPU) [21], poly(butylene succinate) (PBS) [22] and polyethylene glycol (PEG) [23,24] and so on. Li [20] synthesized the crosslinked polyurethane (CPU) through in-situ polymerization, and combining with biological phosphorus flame retardant (PA-HDA) to prepare the PLA composites. The elongation at break and the impact strength of the flame-retardant PLA were increased by 26.6% and 2.8%, respectively, compared with the neat PLA, and passed the UL94 V-0 level with a LOI value of 26%. It is also found that the properties of PLA composites is dependent on the molecular weights of PEG. Zhang [25] prepared FRPLA composites by combining PEG-2000 or PEG-4000 with ammonium polyphosphate (APP). Results showed that 10 wt% PEG-4000 could transform the material from brittle fracture (10% PEG-2000) into ductile fracture, and could pass the UL 94 V-0 level with a LOI value of 27.4%.

As a common reinforcing material, natural fibers are also used to enhance the mechanical properties of flame-retardant PLA materials [26,27]. Commonly used fibers mainly include hemp fiber [28,29], wood fiber [30], bamboo fiber [31] and stem fiber [32]. Hemp fiber has the longest fiber length among natural fibers and has the characteristic of high strength, which makes it popular among researchers. Zhu [33] used microcrystalline cellulose (MCC) extracted from bamboo powder as the biobased carbon source of the expansion system, and prepared MA-MCC by grafting it with methacrylic acid (MA) through graft polymerization. The results showed that the LOI of the PLA composites with 3% MA-MCC and 7% APP could reach 26.8% and pass the UL94 V-0 level, the non-notch impact strength and the Young’s modulus of the PLA composites were improved to 8.16 kJ/m^2^ and 1612.8 MPa (pure PLA: 7.71 kJ/m^2^ and 1245 MPa), respectively.

In addition, some researchers found that some flame retardants with nucleation effect [34,35,36] and core-shell structure [37,38,39] could effectively improve the overall performance of PLA. Ran [40] coated the APP with polyborosiloxane (BSI) to obtain the microencapsulated APP(BSI-APP), and applied in PLA matrix. Results showed that the tensile strength and elongation at break of PLA composites containing only 5% BSI-APP were still better than the pure PLA with the UL94 V-0 level and LOI value of 26.7%. In addition, the comprehensive performance of PLA composites could be improved by some nano-fillers [41,42]. The mechanical properties of polymer nanocomposites are determined by various factors, including the interface bonding force between the nano-fillers and PLA, the modification, size and morphology of the nano-fillers, etc.

Although adding toughening agent and natural fiber can improve the mechanical properties, the addition of flammable components will raise the concern of the flame retardant properties. Recently, a new method of construction of cross-linked structure in flame-retardant PLA was developed to improve the mechanical properties without affecting its flame retardant properties [43,44,45,46,47,48]. The preparation method is simple, and the PLA material can be endowed with good mechanical properties with low amount of cross-linked component. For example, Wen [49] prepared PLA-MA materials with low molecular weight by adding 0.5 wt% DCP and 2 wt%

MA. Then functionalized nanosized carbon black (CB) with active hydroxyl groups (CB-g-DOPO) was applied to improve the flame retardant properties. The polycondensation reaction between MA and CB-g-DOPO can form a cross-linked network structure. Results showed 10 wt% CB-g-DOPO/PLA-MA had a LOI value of 29.3%, and pass the UL94 V-0 level. The pk-HRR was reduced by 50% compared to pure PLA. In the meantime, the elongation at break and impact strength were increased by 870% and 250%, respectively. Xu [50] used triglycidyl isocyanurate (TGIC) as the micro-cross-linked agent, which was added into PLA matrix combined with a P/N structure flame retardant. The results showed that the mechanical properties of flame-retardant PLA materials were similar to that of pure PLA samples by adding only 0.1 wt% TGIC. In addition, the composites could pass the UL94 V-0 level with a LOI value of 30.4%.

In the previous study, we applied intumescent flame retardant in PLA and found that it can greatly improve the flame retardancy of PLA composites and inhibit the dripping, but the major drawback is the adverse impact of the mechanical properties of PLA composites. In order to effectively improve the mechanical properties and flame retardant properties of PLA at the same time, we tried to add different synergists to PLA. It was found that the addition of ADR and nano ZnO could balance the mechanical properties and the flame retardant performance of the flame-retardant PLA. We tried to add different synergists to PLA [51,52]. However, nano ZnO is not recommended to be used in biodegradable plastic PLA because it is toxic to organisms. Therefore, DCP and TAIC were used to construct a crosslinking structure. In previous studies, different proportions of DCP and TAIC were used to determine whether they have synergy effect and the best proportion of them in flame-retardant PLA composites (the data were shown in the abbreviations). In this research, a small amount of DCP and TAIC (less than 1.5 wt%) with a specific proportion, which exert PLA with the best comprehensive performance, was selected to form a cross-linking structure in the flame-retardant PLA. Meanwhile, the control samples were set to study the influence of crosslinking structure on the comprehensive performance of PLA. In addition, the synergistic effect mechanism between DCP and TAIC was further studied.

## 2. Experimental

### 2.1. Materials

PLA resin (2003D) was supplied by Natural Works Company (Blair, NE, USA), and DCP (dicumyl peroxide) was provided by Hubei Kangdis Chemical Co., Ltd (Wuhan, Hubei Province, China). The TAIC (Triallyl isocyanate) was kindly provided by Beijing Banxia Technology Development Co., Ltd (Beijing, China). The ammonium polyphosphate (APP, n > 1000) was supplied by the Hangzhou JLS Flame Retardants Chemical Co. Ltd. (Hangzhou, Zhejiang Province, China). The triazine was purchased from Brother Enterprises Holding Co. Ltd. (Shouguang, Shandong Province, China).

### 2.2. Preparation of PLA Composites

**Figure 1:**
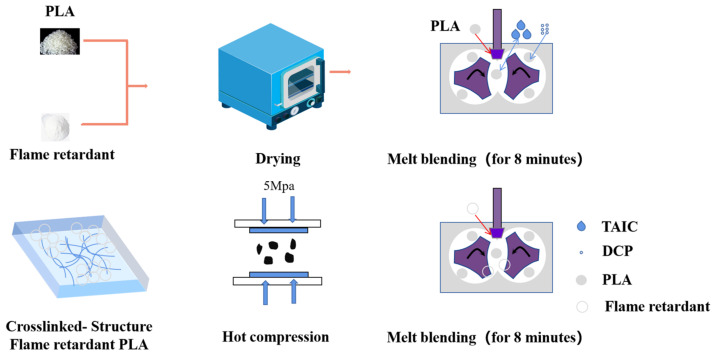
The flow chart of the processing process.

First of all, all raw materials were dried in a vacuum drying oven at 120 °C for 6 h before use. The neat PLA was mixed with DCP, TAIC and DCP/TAIC at a roll speed of 60 rpm for 8 min at 190 °C with a torque rheometer. Then the APP and triazine flame retardants were mixed and added into the torque rheometer with the same process. After mixing, the sample was transferred to the mold and preheated at 190 °C for 6 min, then pressed at 10 Mpa and cooled for 5 min to obtain the standard sample. The proportion of PLA composite material is shown in Table 1. The flow chart of the processing process is shown in Figure 1.

### 2.3. Characteristic

The gel content of PLA composites were determined by chloroform Soxhlet extraction method. First, the material was placed into the chloroform at 55 °C for 8 h, and then filtered, the filter residue was retained, and dried at 80 °C for 6 h to remove the residual chloroform in the material. The experiment was repeated for three times, and the calculation formula was shown in Equation (1). Where *W*_z_, *W_d_* means the total mass and the dissolved mass, and *V*_i_ represents the proportion of the insoluble part of FRPLA.

(1) $$Gelcontent(wt\%)=\frac{W_{z}-(W_{z}\times V_{i}+W_{d})}{W_{z}}\times 100\%$$

The Waters (Waters1515, Milford, CT, USA) gel permeation chromatography (GPC) was used to analyze the molecular weights. In addition, chloroform was the mobile phase.

The MP993A instrument was used to test the melt mass-flow rate (MFR) according to standard ASTM D1238. The load was 2.16 kg with a temperature of 190 °C and a melting time of 180 s. Each group was sectioned 10 times, with an interval of 10 s.

The Phenom Pro scanning electron microscope (Phenom World, Eindhoven, Netherlands) was used to analyze the impact fracture surface morphology after metal spraying with a voltage of 5 kV. And energy spectrum analysis (EDX) was carried out for the impact fracture under 15 kV voltage.

The tensile strength test was performed on the CMT6104 universal testing machine (MTS system, Shanghai, China) according to ISO 527-1 at a tensile rate of 5 mm/min. The results were the average of 5 replicates.

Non-notching impact tests were carried out on XJZ-50 digital impact testing machine (Chengde Testing Machine, Chengde, Hebei Province, China) in accordance with ISO 179-1, using 2J pendulum. The results were averaged over the five tests.

The FTT 0082 instrument (Fire Testing Technology, East Grinstead, West Sussex, UK) was used to test the UL94 vertical combustion test according to ASTM D3801 test standard with a sample size of 130 × 13 × 3.2 mm.

The cone calorimeter test was carried out on the cone calorimeter (Fire Testing Technology, East Grinstead, West Sussex, UK) under a heat flux of 50 kW/m^2^ according to the ASTM ISO 5660 with a sample dimension of 100 × 100 × 3 mm). Every sample was repeated three times.

The limiting oxygen index (LOI) values were studied by the FTT Dynisco LOI instrument (Fire Testing Technology, East Grinstead, West Sussex, UK) according to ASTM D 2863-97 with specimen dimension of 130 × 6.5 × 3.2 mm.

The Phenom Pro scanning electron microscope (Phenom World, Eindhoven, Netherlands) was used to analyze the micromorphology images of the residues after cone calorimeter test under the high vacuum conditions with a voltage of 5 kV.

The thermal stability of PLA composites was obtained using the thermogravimetric analyzer-TGA(STA 8000, PerkinElmer, Waltham, MA, USA) in the temperature range of 50 to 800 °C at a rate of 20 °C/min under N_2_ atmosphere. The experiment was repeated three times.

The crystallization and melting behaviors of different polylactic acid samples were obtained under N_2_ atmosphere by Q20 differential scanning calorimeter (TA Instruments, New Castle, DE, USA). Each sample was about 5–8 mg, heated to 190 °C at 20 °C/min and stored for 5 min to remove the previous thermal history. The samples were then cooled to 20 °C at a cooling rate of 10 °C/min and then heated to 180 °C at a heating rate of 10 °C/min to record crystallization and melting behavior.

## 3. Results and Discussion

### 3.1. Verification of Cross-Linked Network Structures

**Figure 2:**
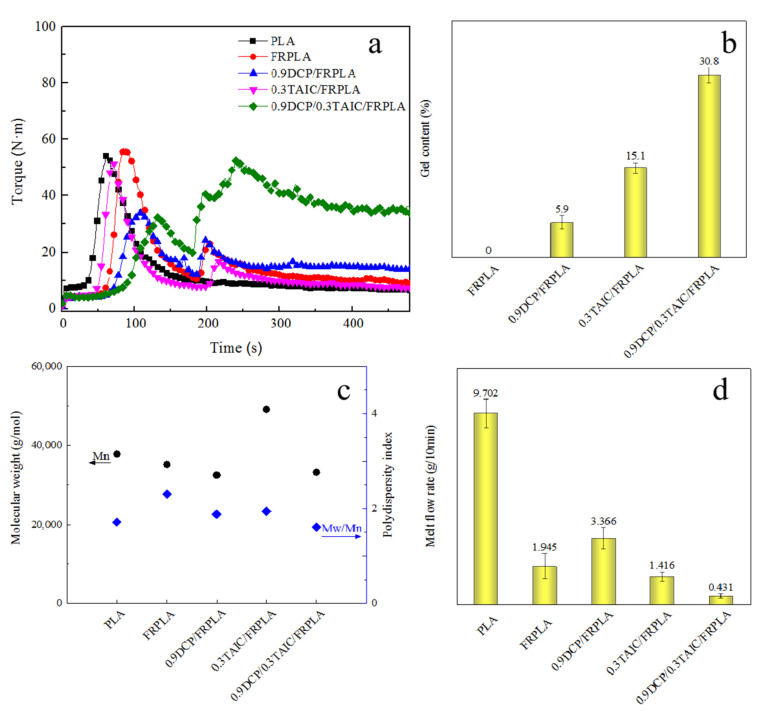
Characterization of crosslinked structures: (**a**) Torque curve; (**b**) gel content; (**c**) molecular weight; (**d**) MFR.

In this section, torque test, gel content test, molecular weight test and MFR test were used to characterize the cross-linked structure. The results are shown in Figure 2, where Figure 2a is the torque test curve, Figure 2b is the gel content test result, Figure 2c is the molecular weight test result, and Figure 2d is the melt flow rate test result.

The torque curve during processing can reflect the formation of cross-linked structures in the PLA composites. As can be seen from Figure 2a, there were two peaks in each torque curve, which was due to the twice addition of the raw material. The flame retardants were added after three minutes. It was not hard to find that the torque at the end of blending was only 6.1 N·m. The final torque of FRPLA was shown to be 8.7 N·m, because the addition of flame retardant as filler increased the viscosity of the melt [53]. It was worth mentioning that when DCP and TAIC were added at the same time, the final torque of 0.9DCP/0.3TAIC/FRPLA reached 35.4 N·m, which verified the formation of the cross-linked structure. In contrast, the final torque of 0.9DCP/FRPLA reached 13.5 N·m, and the overall change was not obvious after the addition of TAIC alone. It implied the synergistic effect between DCP and TAIC in inducing the formation of a cross-linked structure.

In order to further verify the existence of cross-linked structure, the gel content was tested. The test results were shown in Figure 2b. The gel content of 0.9DCP/FRPLA and 0.3TAIC/FRPLA were 5.9% and 15.1%, respectively, which indicated that DCP or TAIC alone could form a certain cross-linked network structure. With the addition of DCP and TAIC in the meantime, the gel content of 0.9DCP/0.3TAIC/FRPLA reached 30.8%. It implied the synergistic effect between DCP and TAIC in inducing the formation of a cross-linked structure.

The molecular weight and the polydispersity index of pure PLA and PLA composites was shown in Figure 2c. As shown in Figure 2c, the M_n_ of pure PLA was 37,810 g/mol. With the addition of FR, the M_n_ of FRPLA decreased to 35,180 g/mol, indicating that the introduction of FR would lead to the degradation of PLA. When 0.9 wt% DCP was added, the M_n_ of 0.9DCP/FRPLA was reduced by 7.5% (32,490 g/mol). This could be ascribed as the introduction of DCP breaking the PLA chain segment and forming the free reactive sites, which resulted in the self-crosslinked phenomenon of PLA chain segment and the short PLA chain, resulting in the decrease of the molecules weight of 0.9DCP/FRPLA. The addition of 0.3 wt% TAIC made the M_n_ of 0.3TAIC/FRPLA increased by 39.6% (49,100 g/mol), which meant that TAIC can increase the molecular weight of PLA and promoted the formation of long chain structure of PLA. When DCP and TAIC were added at the same time, the M_n_ of 0.9DCP/0.3TAIC/FRPLA was 33,220 g/mol. It indicated that the M_n_ of the remaining molecules except cross-linked structure had the similar molecular weight as 0.9DCP/FRPLA because the crosslinked structure could not be filtered in molecular weight test, and only the remaining molecules were analyzed. The results of molecular weight analysis implied that DCP could trigger PLA to form active point in the reaction and reduce the molecular weight of the material, while TAIC could react with PLA as cross-linking site to form long chain structure and cross-linked structures.

The melt flow rate can also confirm the formation of cross-linked structure. When the cross-linked structure was formed in the system, the MFR of the material decreases. The MFR results were shown in Figure 2d. The MFR of pure PLA was 9.702 g/10 min. After the addition of FR, the MFR decreased by 79.9% (1.945 g/10 min). The addition of DCP increased the MFR of PLA composites to 3.366 g/10 min, because the addition of DCP facilitate the PLA chain breaking into short chain. Although there were still some cross-linked structures presented, the overall fluidity of PLA composites was greatly improved compared with FRPLA. The reason can be explained as on the one hand, there were only a few amounts of the crosslinked network structures formed, on the other hand, the movement of the generated small molecular chain segments can effectively improve the mobility of PLA. With the addition of TAIC, the gel content of 0.3wtTAIC/FRPLA increased and long chain structure was formed, which in turn led to the decrease of its fluidity. When DCP and TAIC were added in the meantime, the MFR was only 0.431 g/10 min. It was because the gel content of 0.9DCP/0.3TAIC/FRPLA was the highest of all the PLA composites, which resulted in the lowest fluidity of the composite.

The result of the torque test, gel content test, molecular weight test and MFR test proved the formation of the crosslinking structure in PLA matrix. When DCP and TAIC were added at the same time, the changes in torque, the increase in gel content and the decrease in MFR showed that DCP and TAIC had a synergistic crosslinking effect, which effectively improved the composition of crosslinking. Therefore, the influence of the crosslinked structure on the comprehensive performance of PLA were studied.

### 3.2. The Micromorphology of the Fracture Surface

**Figure 3:**
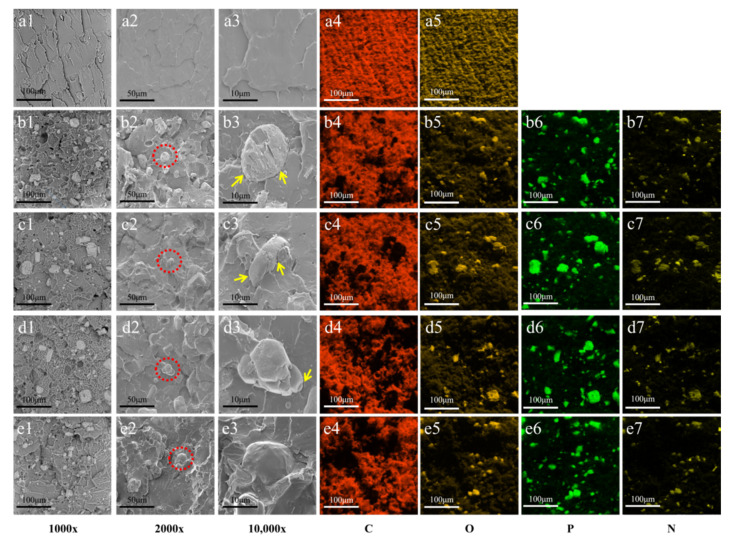
SEM photos of the impact fracture surface: (**a**) PLA; (**b**) FRPLA; (**c**) 0.9DCP/FRPLA; (**d**) 0.3TAIC/FRPLA; (**e**) 0.9DCP/0.3TAIC/FRPLA.

In order to explore the influence of cross-linked structure on the microstructures of the flame-retardant PLA composites, the morphology, composition and dispersion of elements for impact fracture surface were characterized by SEM. The obtained morphology images were shown in the first three columns of Figure 3, and the composition and dispersion of element under 1000× were exhibited in the last four columns, in the order C, O, P and N. To achieve high resolution of the micromorphology display of fracture surface, the SEM was selected at 1000×, 2000× and 10,000×, where the photos of 10,000× are the enlarged photographs of the highlighted position shown by the red circle in the photos of 2000×.

As shown in Figure 3(a1,a2), the surface of PLA was smooth. The addition of flame retardant led to the formation of sea-island structure in PLA matrix (Figure 3(b1,b2)), in which FR acts as the dispersed phase and PLA matrix acts as the continuous phase. The interface between the dispersed phase and the continuous phase was very clear between the flame retardant and PLA matrix, which was marked with yellow arrow. It indicated that the adhesion between the flame retardant and PLA was poor, and possibly resulted in a significant decrease in the mechanical properties of FRPLA. With the addition of DCP (Figure 3(c1–c3)) or TAIC (Figure 3(d1–d3)) alone, it can be observed that the interface between two phases was indistinct (yellow arrow), and the gaps were not obvious compared with that of FRPLA, which indicated that the formation of cross-linked structure can strength the adhesion between the flame retardant and PLA. When DCP and TAIC were added to the matrix at the same time, it was not difficult to find that the gaps between two phases disappeared, and a layer of PLA matrix was coated on the surface of the flame retardant particles (Figure 3(e3)), which indicated that the high cross-linked structure can further improve the adhesion between the dispersed phase of flame retardant and the PLA matrix phase. This effect also suggests the improvement of the adhesion would promote the mechanical properties of PLA composites. The reason why the adhesion between flame retardant domain and PLA matrix increased was that the reactive groups such as NH_2_ on the flame retardant could react with PLA, DCP and TAIC, so that PLA could be coated on the surface of the flame retardant and has strong interfacial adhesion.

In addition, it can be seen from the distribution of phosphorus and nitrogen element that the distribution of phosphorus was more uniform without cross-linked structure (FRPLA). When there was cross-linked structure, especially DCP/FRPLA and TAIC/FRPLA, the distribution of phosphorus element was not uniform anymore, and some agglomerations can be observed. The dispersion of nitrogen element also showed the similar trend. It suggested that the presence of cross-linked structure could lead to partial aggregation of flame retardants, which can affect the effect of flame retardant to certain extent.

The result of SEM and EDX showed that the existence of crosslinked structure can effectively improve the impact performance of PLA composites. Meanwhile, the existence of crosslinked structure could also lead to the aggregation of some flame retardants, and whether this would have an impact on the flame retardant performance needs to be further tested.

### 3.3. The Mechanical Properties of PLA and Its Composites

**Figure 4:**
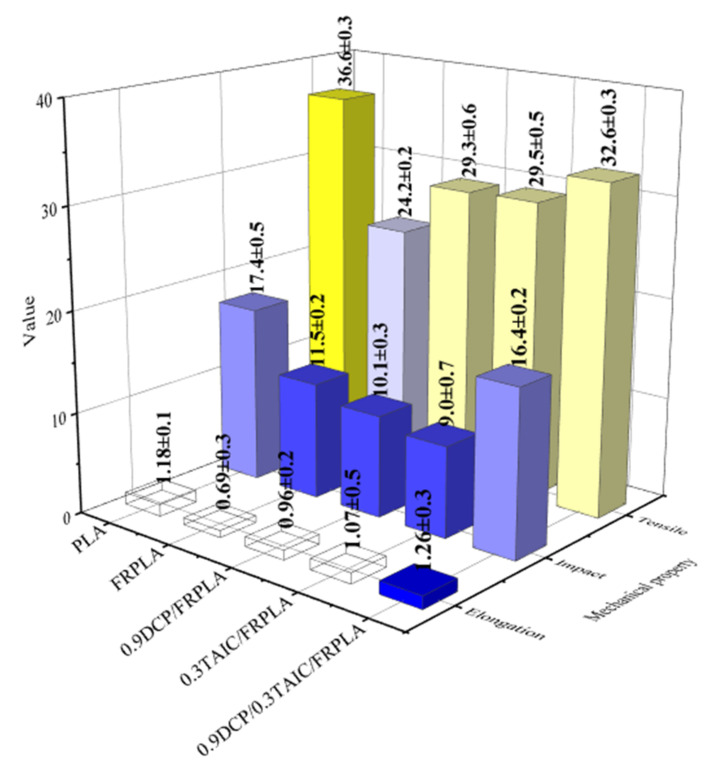
The mechanical properties of pure PLA and PLA composites.

To investigate the effect between the formation of cross-linked structure and the mechanical properties of the PLA composites, impact strength test and tensile strength test were carried out. The test results are shown in Figure 4.

Compared to pure PLA, the tensile strength, elongation at break and impact strength of FRPLA all decreased by 33.9%, 41.5% and 40.0%, respectively. This indicated that the addition of FR will deteriorate the mechanical properties of PLA matrix. After the addition of DCP or TAIC alone, the tensile strength and the elongation at break were improved but not for the impact strength, which could be interpreted as both of DCP/FRPLA and TAIC/FRPLA system have formed the cross-linked network. The formation of the cross-linked network finally affected the distribution of flame retardants, resulting in uneven distribution of flame retardants, thus leading to a decrease in impact strength. It was also worth mentioning that the tensile strength, the elongation at break and the impact strength of DCP/TAIC/FRPLA is 34.8%, 82.6% and 42.9% are higher than that of FRPLA, respectively, and the elongation at break of DCP/TAIC/FRPLA was higher than that of pure PLA.

The mechanical properties test results showed that when DCP and TAIC were added at the same time, the mechanical properties improved significantly and were close to that of pure PLA. Compared with the ADR/nano ZnO system [51], the mechanical properties of the two system are similar to those of pure PLA, which means that both crosslinked structures can achieve excellent performance. In addition, the study of Zhang [54] showed that the mechanical properties of PLA composites decreased significantly after the addition of flame retardants. The existence of crosslinking network can make the material withstand greater stress.

### 3.4. The Flame Retardant Performance of PLA and Its Composites

**Figure 5:**
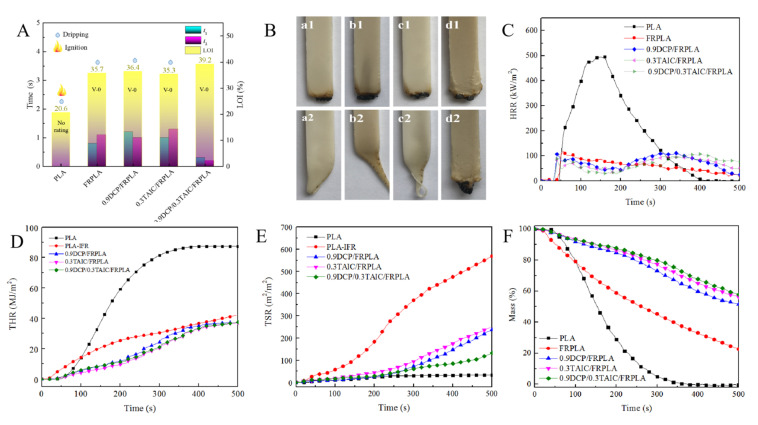
The flame retardant properties of PLA and PLA composites: (**A**) LOI and UL 94 vertical burning test; (**B**) digital image of composite UL94 sample (a: FRPLA, b: 0.9DCP/FRPLA, c: 0.3TAIC/FRPLA, d: 0.9DCP/0.3TAIC/FRPLA); (**C**) HRR curves; (**D**) THR curves; (**E**) TSR curves; (**F**) mass loss curve.

The above results verified the synergistic effect between DCP and TAIC in forming the cross-linked structure and the synergistic effect could improve the mechanical properties of FRPLA. Therefore, in this section, the limiting oxygen index (LOI), the UL94 vertical burning test and cone calorimeter test were carried out to study the influence of cross-linked structure on the flame retardant performance of PLA and its composites. The test results were shown in Figure 5. The specific data of cone calorimeter test were summarized in Table 2.

As shown in Figure 5A, the LOI of pure PLA was 20.6%, and it burned completely with severe dripping in vertical burning test. With the addition of FR, the oxygen index reached 35.7% and passed the UL 94 V-0 level. Although there is dripping, it does not ignite the absorbent cotton. When DCP or TAIC was added separately, the PLA composites could still pass the UL 94 V-0 level with slight drip and little change in oxygen index. It was worth noting that when DCP and TAIC were added simultaneously, the LOI value of 0.9DCP/0.3TAIC/FRPLA sample increased to 39.2%. During vertical burning test, there was no dripping and the values of *t*_1_ and *t*_2_ were much smaller than that of other composites, which indicated that a higher content of cross-linked structure can effectively inhibit the combustion and dripping of PLA.

Digital images of the PLA and its composites after first and second ignition during vertical burning test were collected, and shown in Figure 5B a1, b1, c1 and d1 are the images after first ignition, while a2, b2, c2 and d2 are the photos after second ignition. It exhibited that all the flame retardant samples can form an effective protective carbon layer during the first combustion. However, in the second combustion, it can be clearly seen that a2, b2 and c2 all have a dripping behavior, which occurred after the second ignition. The complete coronal carbon layer only remained on the ignition end of d2, indicating clearly that the cross-linked structure restricted the dripping behavior. All the above flame retardant test results again supported the hypothesis that the cross-linked structure generated by the synergistic effect of DCP and TAIC can effectively improve the flame retardant performance of FRPLA. At present, some of the flame retardancy of PLA are difficult to completely inhibit dripping. For example, Liu [55] synthesized a new flame retardant and added into PLA, although the flame performance was excellent, it still could not completely hinder the dripping phenomenon.

The flame retardant properties of PLA and PLA composites were investigated by the cone calorimeter test, and the results, including the time to ignition (TTI), heat release rate (HRR), total heat release (THR), total smoke production (TSP) and mass loss, etc., are shown in Table 2. In addition, the HRR curves (Figure 5C), THR curves(D), TSR curves(E) and mass loss curves(F) as a function of combustion time for pure PLA and PLA composites are displayed in Figure 5.

As shown in Figure 5C, the pk-HRR of pure PLA reached 494 kW/m^2^, which was the highest among all the samples. With the addition of flame retardant, the pk-HRR of FRPLA decreased to 119 kW/m^2^, indicated that FR effectively suppressed combustion. With the addition of DCP, the HRR curve showed two combustion peaks, which was different from FRPLA. Within the range of the first peak (within 40~200 s), HRR decreased gradually, which was possibly correlated to the formation of a protective carbon layer. As the combustion continued, the HRR increased from 200 s, then the second peak was formed, which can be attributed to the rupture of the initial carbon layer resulted from the uneven distribution of flame retardants. This phenomenon of two combustion peaks can also be seen in the 0.3TAIC/FRPLA and 0.9DCP/0.3TAIC/FRPLA composites. The pk-HRR decreased by 6.7%, 18.5% and 9.3%, respectively, after the addition of DCP, TAIC and DCP/TAIC system. The cross-linked network structure showed little effect on the peak heat release rate. The trend of peak heat release rate is the same as that of molecular weight. The smaller the molecular weight, the higher the pk-HRR.

As shown in Figure 5D, the THR of pure PLA is 87 MJ/m^2^, as an evidence of complete combustion of pure PLA. With the addition of FR, the THR decreased to 42 MJ/m^2^. The introduction of flame retardant slowed down the combustion of PLA matrix. Compared to FRPLA, the THR decreased slightly with the addition of DCP, TAIC and DCP/TAIC. This phenomenon could be attributed to two factors: (a) The protective effect of carbon layer containing cross-linked structure was beneficial for the improvement of the flame retardancy; and (b) the aggregation of flame retardants caused by the cross-linked structure reduced the flame retardant effect. These two kinds of actions were opposite to each other, resulting in only a slight decrease in THR, so as the pk-HRR.

In the TSR curves, the TSR decreased with the increase of gel content. The TSR of 0.9DCP/0.3TAIC/FRPLA was only 140 m^2^/m^2^, which was 75.2% lower than that of FRPLA (563 m^2^/m^2^). It indicated that the formation of cross-linking structure can make more carbon locked in the carbon layer rather than released in the form of smoke. As shown in Figure 5F, the final mass of pure PLA was only 0.2 wt%. The addition of FR aided the formation of the final carbon residue, which increased to 22.6 wt%. With the formation of the cross-linked structure, it can be found that the final mass of 0.9DCP/FRPLA, 0.3TAIC/FRPLA and 0.9DCP/0.3TAIC/FRPLA were all significantly increased to about 45%, which showed that the formation of crosslinking network structure can boost the carbon residue. Cone calorimetry results showed that the formation of crosslinking network structure can make the peak heat release rate and total heat release decrease slightly, but it can greatly lessen the total smoke release and increase the carbon residue.

The test results of flame retardant performance showed that the crosslinked network structure formed by DCP and TAIC reduced HRR and THR, increased the final mass and inhibited the smoke release of PLA during the combustion. These indicated that the existence of crosslinking network was beneficial to the improvement of flame retardant performance. Compared with the ADR/nano ZnO system, the synergistic effect of DCP/TAIC system was more conducive to the reduction of HRR, which meant the cross-linking structure constructed by DCP and TAIC had a greater promotion effect on the flame retardant effect than the ADR/nano ZnO system.

### 3.5. The Structural and Morphology Analysis of Residuary Char of PLA Composites

**Figure 6:**
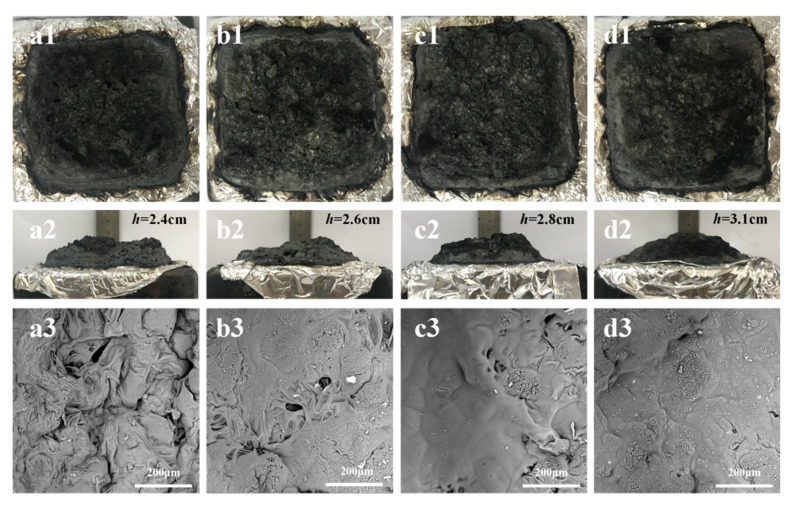
Residual char digital photos and SEM photos (400×) of the residual char after cone calorimeter test: (**a1**–**a3**) FRPLA; (**b1**–**b3**) 0.9DCP/FRPLA; (**c1**–**c3**) 0.3TAIC/FRPLA; (**d1**–**d3**) 0.9DCP/0.3TAIC/FRPLA.

The flame retardant mechanism of PLA composites was studied through the characterization of carbon residue in cone calorimetry test. The macroscopic and microscopic morphology of PLA composites are shown in Figure 6.

It can be seen from Figure 6(a1) that FRPLA showed typical morphology of intumescent carbon layer, and the final height of carbon residue was about 2.4 cm (Figure 6(a2)). After the addition of DCP, TAIC or DCP/TAIC, the surface of carbon layer was still complete and compact. Meanwhile, the height of 0.9DCP/FRPLA, 0.3TAIC/FRPLA and 0.9DCP/0.3TAIC/FRPLA increased to 2.6 cm (Figure 6(b2)), 2.8 cm (Figure 6(c2)) and 3.1 cm (Figure 6(d2)), respectively. The reason was that the higher proportion of cross-linked structure and lower MFR led to the decrease of melt fluidity and the increase of melt strength, which improved the viscosity of the PLA melt during combustion, and the final formation of the intumescent and complete char layer were obtained. Combined with the above test results, it was found that the height of the carbon layer gradually increased with the increasing cross-linked structures, which revealed that the crosslinking structure was beneficial to the formation of intumescent carbon layer, which can protect the underlying matrix during the combustion process. As could be seen from Figure 6(a3–d3), with the addition of FR (Figure 6(a3)), although the carbon layer was generated, there are still high density of holes on the surface of the final char layer. With the introduction of either DCP (Figure 6(b3)) or TAIC alone (Figure 6(c3)), both the number and size of the holes on the surface of the char layer were reduced. When DCP and TAIC were added together, the surface of char residue was compact, complete and holeless (Figure 6(d3)) This was because the formation of cross-linking structure was conducive to improving the melt strength, which made it difficult for the generated gas to break through the carbon layer and form holes.

SEM results of carbon residue showed that the existence of crosslinking structure can promote the formation of carbon layer during combustion, and make the final carbon layer denser, so as to play a better protection effect.

### 3.6. The Thermal Performance of PLA and PLA Composites

**Figure 7:**
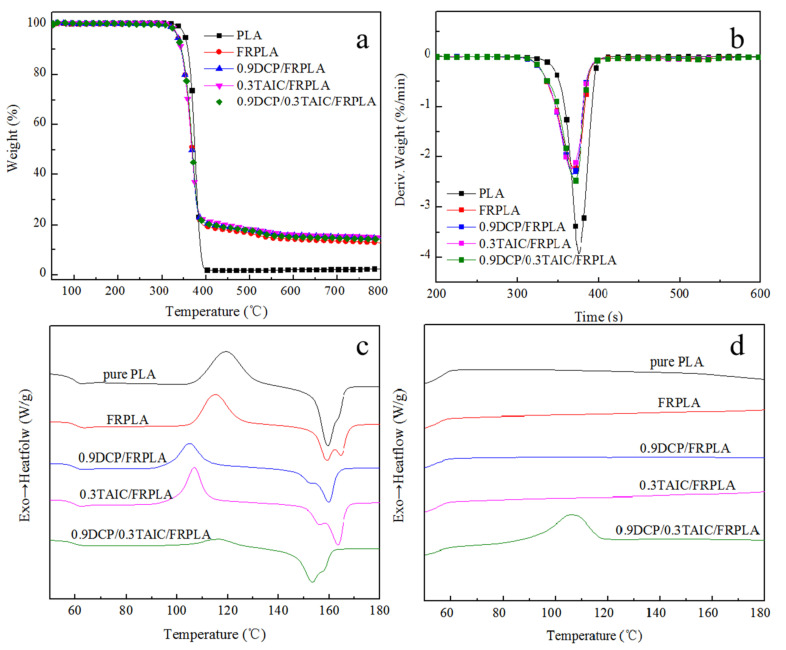
The curves of thermal performance: (**a**) TG curves; (**b**) DTG curves; (**c**) the crystallization curves of the heating process; (**d**) the crystallization curves of the cooling process.

The thermal performance of the studied materials was investigated by means of TG analysis and the DSC under N_2_ atmosphere; the curves of TG and DTG are presented in Figure 7a,b, the crystallization curves of the heating process and the cooling process are shown in Figure 7c,d. In addition, the temperature at 5% mass loss (*T*_onset_), the maximum decomposition temperature (*T*_max_) and the residue (wt%) at 600 °C are shown is Table 3.

As shown in Figure 7a,b, the PLA had a *T*_onset_ of 353 °C with a maximum weight loss occurred at 380 °C. Compared with the pure PLA, the *T*_onset_ and *T*_max_ of the PLA composites containing flame retardant were all declined. The decrease of *T*_onset_s were attributed to the early decomposition of the flame retardant, while the decrease of *T*_max_s could be explained by the fact that the maximum decomposition temperature of the flame retardant was earlier than PLA and the content of flame retardant was high (20%) in the composites. Among them, 0.9DCP/FRPLA showed lower values of *T*_onset_ and *T*_max_, which could be interpreted as that the long PLA chains were broken into short PLA chain thanks to the catalysis of DCP, and yielded a lower decomposition temperature. The addition of TAIC and DCP to small molecules did not reduce the decomposition temperature, which was due to the cross-linked structure formed from the complete reaction between them and PLA. Besides, the residual char amount at 700 °C of 0.9DCP/FRPLA, 0.3TAIC/FRPLA and 0.9DCP/0.3TAIC/FRPLA were all higher than that of FRPLA, indicating that the existence of a cross-linked structure was a benefit for the formation of final mass.

Figure 7c,d shows the crystallization profiles of the heating process and the cooling process of PLA. The glass transition temperature (*T_g_*), melting point (*T_m_*), cold crystallization temperature (*T_c_*), the heat of fusion (${\Delta H}_{m}$), the enthalpy of cold crystallization (${\Delta H}_{c}$) and the corresponding crystallinity (*X_c_*) of PLA and PLA composites are shown in Table 4, and the calculation formula of *X*_c_ of all the samples was as follow:

$$X_{c}=\frac{{\Delta H}_{m}-\Delta H_{c}}{{\Delta H}_{m(PLA)}^{0}\times W_{\left(PLA\right)}}\times 100\%$$

According to Figure 7c, the addition of 0.9 wt% DCP, 0.3 wt% TAIC or 0.9 wt% DCP/0.3 wt% TAIC had no significant effect on *T_g_* of PLA composites, while it had a large impact on *T_m_*. The *T_m_* of FRPLA and 0.9DCP/FRPLA were around 160 °C, the same as the pure PLA. However, with the introduction of TAIC, it could be found that the *T_m_* of 0.3TAIC/FRPLA increased to 163 °C, which was attributed to the increase of molecular weight due to the chain extension effect of TAIC. As a result, the movement ability of the whole segment decreased and the *T_m_* increased. When DCP and TAIC were added in the meantime, the *T_m_* decreased significantly. This may be due to the formation of cross-linked and branched structures after the addition of DCP and TAIC, which leads to the change of crystal form of PLA and the appearance of imperfect crystallization, corresponding to a lower melting point.

The most obvious difference in Figure 7d was the appearance of the crystallization peak in the crystallization curve of 0.9DCP/0.3TAIC/FRPLA, around 107 °C, which meant that the crystallization capacity of PLA material can be greatly improved by the addition of DCP and TAIC. The reason was that the introduction of DCP and TAIC led to the formation of branched structures of PLA in the system, which promoted the heterogeneous nucleation and crystallization rate of PLA [56]. Improvement of the crystallization rate of PLA was a hotspot that researchers have been studying for a long time, this study provides a new method to improve the crystallization capacity of PLA. Meanwhile, the improvement of the crystallization rate and crystallization ability of the material also significantly improved the crystallinity of PLA composites. Compared with the pure PLA, the *X_c_* of 0.9DCP/0.3TAIC/FRPLA increased from 2.1% to 22.6%.

The TGA test results showed that the introduction of cross-linked structure can effectively improve the formation of final carbon residue. DSC test results showed that the addition of 0.9% DCP and 0.3% TAIC could increase the crystallization rate and promote the crystallization of PLA.

### 3.7. The Work Mode of DCP and TAIC in Flame-Retardant PLA

**Figure 8:**
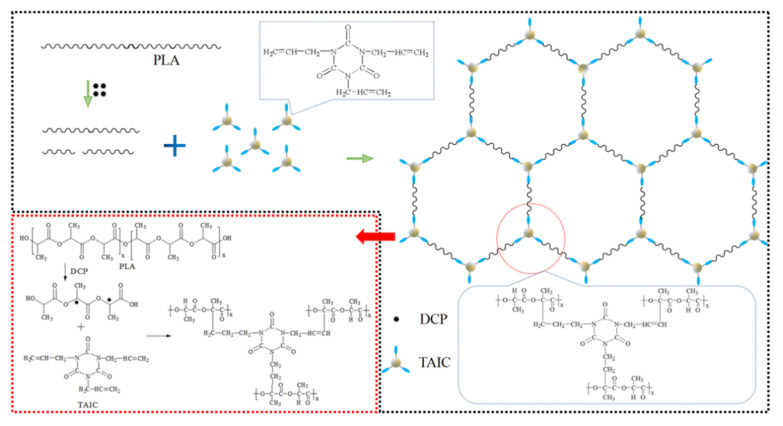
The formation of cross-linked structure in flame-retardant PLA composite under the action of DCP and TAIC.

Under the action of DCP and TAIC, crosslinked structures were formed in flame-retardant PLA composite. The reaction mechanism of DCP and TAIC was shown in Figure 8. First, DCP initiator decomposes and produces free radicals when the temperature raises to about 120 °C. Then the hydrogen atoms on the adjacent carbon atoms of the ester bond on the PLA molecular chain are adsorbed by free radicals, thus free radicals are generated on the PLA chain segments. After that, the grafting copolymerization reaction between the double bond of TAIC and the free radicals on the PLA segment occurs. The reaction continues and finally forms the cross-linked network structures. The cross-linked network structures can endow PLA with excellent flame retardant and mechanical properties.

## 4. Conclusions

In this study, 0.9%DCP and 0.3%TAIC were added to form a cross-linking structure in the flame-retardant PLA composites. The crosslinked structure, mechanical properties, flame retardancy and thermal properties of PLA, FRPLA, 0.9%DCP/FRPLA, 0.3%TAIC/FRPLA and 0.9DCP%/0.3%TAIC/FRPLA composites were tested and characterized.

(1) Firstly, the crosslinked structure was proved to be formed. Compared with FRPLA, the increase (307%) of torque and the decrease (77.8%) of melt flow rate verified that the addition of 0.9%DCP and 0.3%TAIC could form the effective crosslinked structure. The results of gel content test showed that there was a synergistic cross-linking effect between DCP and TAIC, and the gel content of 0.9DCP/0.3TAIC/FRPLA reached 30.8%, which was the highest among all the PLA composites. Compared with FRPLA, the molecular weight of 0.9DCP/FRPLA decreased by 7.5%, and the molecular weight of 0.3TAIC/FRPLA increased by 39.6%, which verified the catalyzed chain breaking effect of DCP and the chain extender effect of TAIC.
(2) Secondly, the formation of crosslinked network structure could improve the mechanical properties. The tensile strength, elongation at break and the impact strength of 0.9DCP/0.3TAIC/FRPLA increased by 34.8%, 82.6% and 42.9%, respectively, which were the highest among the PLA and FRPLA composites.
(3) Thirdly, the formation of crosslinked network structure could improve the flame retardant performance. Results showed that 0.9DCP/0.3TAIC/FRPLA had the highest LOI value (39.2%) and passed the UL94 V-0 grade without dripping. Compared with FRPLA, the HRR of 0.9DCP/0.3TAIC/FRPLA was reduced by 9.3%, and the final carbon residue increases from 22.6% to 44.8%. This is because the cross-linked structure can effectively form an effective protective carbon layer in the cone calorimeter test.
(4) Finally, the TGA test results showed that the introduction of cross-linked structure can effectively improve the formation of final carbon residue. The DSC test results showed that a obvious crystallization peak can be observed on the cooling curve of 0.9DCP/0.3TAIC/FRPLA at the cooling rate of 10 °C/min, and the crystallinity was as high as 22.6%. It indicated that the addition of 0.9% DCP and 0.3% TAIC could increase the crystallization rate and promote the crystallization of PLA.

## Figures and Tables

**Table 1:** Formulations of PLA composites.

Number	Sample	PLA (g)	Triazine		APP		DCP		TAIC	TAIC
			(g)	(wt%)	(g)	(g)	(g)	(wt%)	(g)	(wt%)
1	PLA	200	0	0	0	0	0	0	0	0
2	FRPLA	160	8	4	32	0	0	0	0	0
3	0.9DCP/FRPLA	160	8	4	32	0	0	0.9	0	0
4	0.3TAIC/FRPLA	160	8	4	32	0.48	0.48	0	0.48	0.3
5	0.9DCP/0.3TAIC/FRPLA	160	8	4	32	0.48	0.48	0.9	0.48	0.3

**Table 2:** Combustion parameters of pure PLA and PLA composites during cone calorimeter test.

Sample	TTI (s)	pk-HRR (kW/m^2^)	av-HRR (kW/m^2^)	THR (MJ/m^2^)	av-MLR (g/s)	Final Mass (wt%)	TSR (m^2^/m^2^)
PLA	39	494	249	87	0.111	0.2	30
FRPLA	28	119	43	42	0.034	22.6	563
0.9DCP/FRPLA	26	111	64	37	0.040	44.9	241
0.3TAIC/FRPLA	27	97	57	38	0.033	45.2	244
0.9DCP/0.3TAIC/FRPLA	30	108	57	38	0.036	44.8	140

**Table 3:** The data of TG and DTG for PLA and PLA composites under N_2_ atmosphere.

Samples	N_2_		
	*T*_oneset_/°C	*T*_max_/°C	Residual at 600 °C (wt%)
PLA	353	380	2.1
FRPLA	337	374	14.2
0.9DCP/FRPLA	336	373	15.8
0.3TAIC/FRPLA	337	374	16.1
0.9DCP/0.3TAIC/FRPLA	337	374	15.7

**Table 4:** The DSC data of PLA and flame-retardant PLA composites.

Samples	*T_g_* (°C)	*T_m_* (°C)	${\Delta H}_{m}$ (J/g)	${\Delta H}_{c}$ (J/g)	*X_c_* (%)	*T*_c_ (°C)
PLA	60.3	159.4	33.1	31.1	2.1	119.2
FRPLA	60.6	159.1	26.1	23.4	3.6	114.9
0.9DCP/FRPLA	60.6	159.8	21.5	16.5	6.6	104.6
0.3TAIC/FRPLA	60.3	163.3	24.9	21.3	4.9	106.9
0.9DCP/0.3TAIC/FRPLA	59.6	153.3	23.6	6.7	22.6	116.6

## Data Availability

The data that support the findings of this study are available from the corresponding authors on request.

## Abbreviations

**Full Name**	**Abbreviation**
Poly(lactic acid)	PLA
Flame-retardant poly(lactic acid)	FRPLA
Thermoplastic polyurethane elastomer	TPU
Crosslinked polyurethane	CPU
Biobased unsaturated polyester	BPU
Poly(butylene succinate)	PBS
Polyethylene glycol	PEG
Ammonium polyphosphate	APP
Microcrystalline cellulose	MCC
Methacrylic acid	MA
Polyborosiloxane	BSI
Carbon black	CB
Triglycidyl isocyanurate	TGIC
Dicumyl peroxide	DCP
Triallyl isocyanate	TAIC
Gel permeation chromatography	GPC
Melt mass-flow rate	MFR
Energy spectrum analysis	EDX
Thermogravimetric analyzer	TGA
Number-average molecular weight	M_n_
Weight-average molecular weight	M_w_
Flame retardant	FR
The limiting oxygen index	LOI
The time to ignition	TTI
Heat release rate	HRR
Total heat release	THR
Total smoke production	TSP
The temperature at 5% mass loss	*T*_onset_
The maximum decomposition temperature	*T*_max_
The glass transition temperature	*T*_g_
Melting point	*T*_m_
Cold crystallization temperature	*T*_c_
The heat of fusion	${\Delta H}_{m}$
The enthalpy of cold crystallization	${\Delta H}_{c}$
The corresponding crystallinity	*X*_c_
